# Novel Advances in the Evaluation and Treatment of Children With Symptoms of Gastroesophageal Reflux Disease

**DOI:** 10.3389/fped.2022.849105

**Published:** 2022-04-01

**Authors:** Rachel Rosen

**Affiliations:** Boston Children’s Hospital, Boston, MA, United States

**Keywords:** gastroesophageal reflux disease, impedance, functional luminal impedance planimetry, endoscopy, prucalopride, proton pump inhibitor (PPI)

## Abstract

Gastroesophageal reflux disease has long been implicated as a cause for multiple pediatric symptoms ranging from abdominal pain and regurgitation to cough and dental erosions. Diagnostic testing has evolved greatly over the last 20 years; initial testing with pH-metry to measure esophageal acid reflux burden has evolved into measurement of both acid and non-acid reflux and liquid and gas reflux. However, measuring reflux burden alone only tells a small part of the GERD story and many symptoms originally thought to be reflux related are, in fact, related to other disorder which mimic reflux. The current paradigm which involves empiric treatment of symptoms with acid suppression has been replaced with early testing for not only gastroesophageal reflux but also for other diagnostic masqueraders. The focus for interventions has shifted away from acid suppression toward motility interventions and includes a greater recognition of both functional and motility disorders which present with reflux symptoms.

## Tests for Reflux Symptoms

Gastroesophageal reflux has been implicated as a cause for wide ranging signs and symptoms in children including regurgitation, epigastric pain, cough, and pneumonias. However, studies using pH probes often fail to show a consistent association with reflux events and signs and symptoms. This lack of association raises the questions that: (1) these signs or symptoms are not acid reflux related or that (2) our current technology lacks the sensitivity to measure esophageal events. With the addition of impedance to pH monitoring (pH-MII), clinicians have gained new insight into the role of non-acid reflux and of gas in the development of pediatric symptoms; up to 89% of reflux episodes in infants and children are non-acidic and up to 2.4% of symptoms are triggered by gas episodes such as supragastric belching (1–3).

However, beyond just further subtyping reflux categories (acid/non-acid, liquid/gas/mixed), clues from standard pH-MII testing can help to identify reflux masqueraders. For example, in patients with a high correlation (e.g., >90% of symptoms are associated with reflux events) between reflux event and typical symptoms such as heartburn, regurgitation, and chest pain, rumination syndrome should be considered. While diagnosed by performing standard high resolution esophageal manometry or 24-h manometry-impedance measurements (4, 5), where high pressure waves are seen emanating from the stomach forcing gastric contents into the stomach, pH-MII clues can support the diagnosis; in addition to a high symptom correlation, a high clustering of symptoms during and immediately following a meal, a high number of full column reflux episodes, a high proportion of symptoms occurring immediately before the esophageal event and a higher prevalence of other functional esophageal disorders (such as supragastric belching which can be seen on the same pH-MII tracings) all support the diagnosis of rumination (6, 7). Making a diagnosis of rumination is critical as the therapies are behavioral interventions such as diaphragmatic breathing rather than the addition or escalation of acid suppression therapy. In addition to rumination, a high symptom correlation may also support a diagnosis of reflux hypersensitivity, where patients do not have pathologic amounts of reflux but they have a high reflux-symptom correlation; in pediatrics, one quarter of children have a diagnosis of reflux hypersensitivity (8). While acid suppression may play a role in symptom management, neuromodulators to reduce pain signaling may also be important. While providing evidence of a positive symptom correlation is important to tailor reflux therapies, proving a lack of correlation can be equally important. For example, a diagnosis of functional heartburn is made when patients have typical reflux symptoms but, at the time of the symptoms, there are no reflux events detected by pH or pH-MII monitoring. Since these symptoms are not triggered by reflux events, clinicians are able to stop prescribing reflux therapies and focus on neuromodulation to address the symptoms.

**FIGURE 1:**
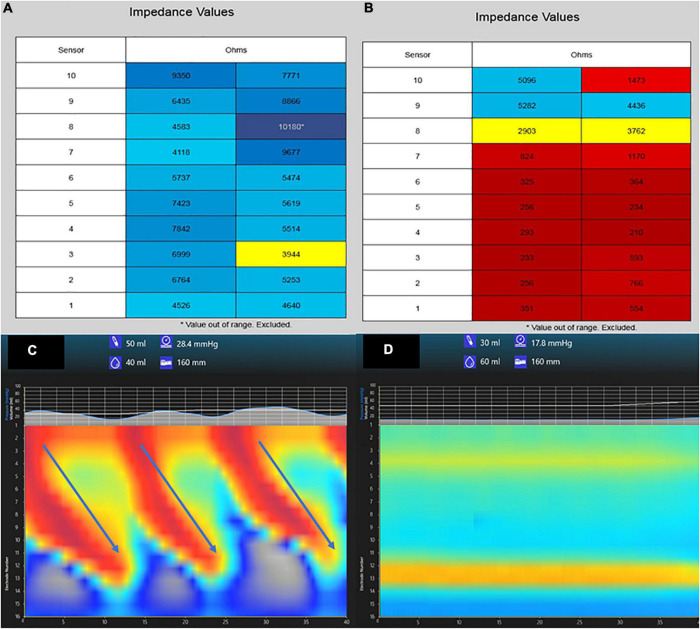
Tracings from mucosal impedance and functional luminal imaging probe (FLIP) technology. A patient with normal esophageal mucosa has high impedance values (>3,000 ohms) at multiple esophageal levels shown in blue **(A)**, whereas a patient with pan-esophageal inflammation has low impedance values (<1,000 ohms) at multiple levels shown in red **(B)**. A FLIP tracing from a patient with normal secondary peristalsis and esophagogastric junction relaxation **(C)** shows repetitive antegrade contractions (RACs, arrows) whereas a patient with absent contractions show no RACs and absent EGJ relaxation **(D)**.

Apart from advances in the interpretation of pH-MII testing, new technologies have provided insight into the risk factors for gastroesophageal reflux and its associated complications. For example, intraprocedural mucosal impedance technology has been used to determine the extent of esophageal inflammation at the time of endoscopy, as a proxy for multiple esophageal biopsies (9–11); while older impedance catheters have been used in pediatric and adult validation studies (10, 12), newer technology to more precisely map the esophagus using impedance strips affixed to an esophageal balloon have been used. Depending on the real-time impedance patterns seen when a balloon with impedance strips is inflated in the esophagus to oppose the strips to mucosa, diagnoses may be made at the time of the index endoscopy and impedance patterns may be followed longitudinally without biopsy; low impedance values signify inflammation and are depicted as a red color and high impedance values signify healthy mucosa and are depicted as a blue color (Figures 1A,B). While not providing different measurements from biopsy in the pediatric population, more precise mapping at multiple esophageal levels might provide some immediate diagnostic clarity with the ultimate goal of reducing pathology costs and reducing time to definitive diagnosis. Currently though, its role in the evaluation of gastroesophageal reflux is not different than multi-level biopsies in children.

**FIGURE 2:**
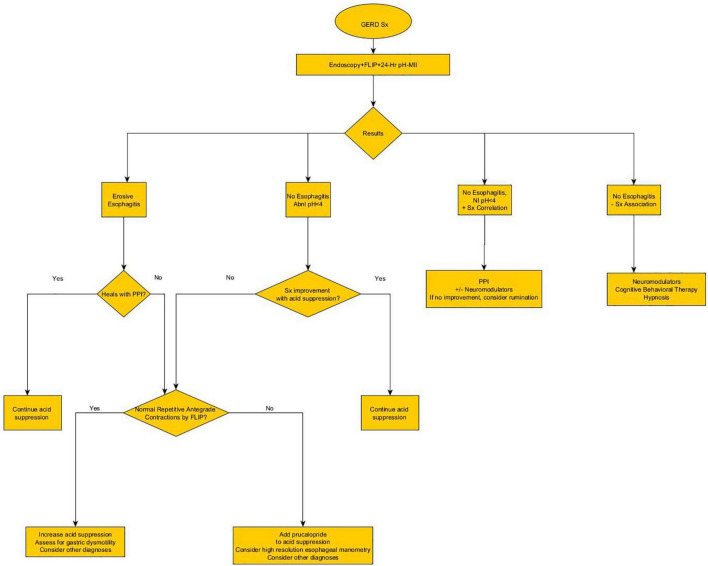
Proposed future algorithm for the diagnosis and treatment of pediatric reflux, incorporating novel technology at the time of the index endoscopy.

A second new technology that provides insight into the mechanisms of reflux is the functional luminal imaging probe (FLIP). FLIP offers additional insight into esophageal motility and distensibility. By inflating a balloon in the esophagus, sensors can measure (1) how distensible the esophagus and esophagogastric junction (EGJ) are and (2) the strength, frequency, and completeness of esophageal contractions seen with secondary peristalsis. Studies of FLIP in patients at risk for reflux have shown that poor peristaltic response to balloon distension predicts abnormal pH-MII testing and this is independent of EGJ distensibility; intact secondary peristalsis (Figure 1C) is needed to effectively clear reflux episodes (13), a finding corroborated with studies of high resolution esophageal manometry and pH-MII testing (14). FLIP also has utility in assessing the post-fundoplication esophagus; patients with poor EGJ distensibility or absent secondary peristalsis (Figure 1D) may have a better therapeutic response to interventions such as dilation or lower esophageal BoTox injections (15–17). FLIP holds the greatest potential in revolutionizing the evaluation of pediatric patients with reflux symptoms. The technology, when performed at the time of index endoscopy, screens for both peristaltic disorders and EGJ outflow disorders which can either masquerade as reflux (i.e., retrograde flow of bolus stasis) or can predispose to poor refluxate clearance. This technology is particularly powerful in pediatrics where performance of esophageal motility studies can be invasive. The potential clinical pathway for pediatric use in the future is shown in Figure 2.

Paralleling the development of novel technologies which are aimed at improving the diagnosis of reflux and its associated triggers, novel therapies which move away from acid suppression have been developed to treat reflux symptoms. This move away from acid suppression is grounded in evidence that shows an increased risk of complications from acid suppression combined with a lack of efficacy in certain high risk populations.

## Proton Pump Inhibitor Risk

While proton pump inhibitors (PPIs) are effective in healing esophagitis and improving symptoms in many patients with typical reflux symptoms, new data has emerged about potential risk. While studies of acid suppression have been shown to alter pediatric and adult infection risk by altering the microbiome, new studies have suggested that viral infection risk may also be increased in adults and children taking PPIs (18–23). Most recently, COVID-19 infection risk and disease severity may be greater in adults and children taking PPIs, a warning to patients with comorbidities that put them at higher risk for more severe disease (24–26). In addition to the newer viral data, studies suggest that PPI use, particularly in early childhood, may increase risk of the development of eosinophilic esophagitis (EoE) and other allergic diseases potentially converting a short-lived disorder (i.e., GERD) to a lifetime disorder (e.g., EoE) (27, 28). Finally, new pediatric data has confirmed what other adult studies have shown, that children exposed to PPIs are at higher risk for bone fractures and this relationship is more significant with cumulative exposure (29, 30). Given this new data, thoughtful prescribing (i.e., lowest dose possible with regular re-evaluation of need) for acid-related disorders is critical. For patients with persistent symptoms despite acid suppression, early testing and consideration of other therapies is needed.

## Medication Therapies Beyond Acid Suppression

Since the only therapy to reduce transient relaxations of the lower esophageal sphincter, the main mechanism of reflux, is baclofen which has significant side effects (31–33), the focus of motility therapies has been to improve esophageal and gastric dysmotility based on the hypothesis that improving esophageal motility results in improved reflux clearance and improving gastric motility may reduce the volume of gastric contents that could be refluxed. While early studies have shown an inconsistent relationship between gastric emptying and reflux burden (34–37), recent data have shown that esophageal and gastric dysmotility may be the biggest predictor of persistent reflux symptoms and complications such as esophagitis in adults and in select high risk pediatric populations such as patients with esophageal atresia or with a history of prior lung transplantation (14, 38–40).

One of the mainstays of motility therapy in pediatrics has been erythromycin, a motilin agonist, which increases antral contractions to improve gastric emptying. While early studies using pH probes in preterm infants failed to show a benefit in reducing acid reflux burden in preterm infants, studies were limited because pH-MII was not used so non-acid reflux episodes, the most common type of reflux in preterm infants, could not be detected (41). However, recently the definitive study in preterm infants was performed showing no benefit of erythromycin in reducing total reflux episodes (acid + non-acid) reflux events, a finding similar to that seen in adults using the macrolide azithromycin (42, 43).

Recognizing the disappointing results of macrolides to reduce the total number gastroesophageal reflux events, other motility agents have been studied. One medication with the most promise because of its effects on both esophageal and gastric motility is prucalopride, a 5-HT4 agonist. While approved as a constipation treatment for adults, the medication has shown significant promise to treat upper tract motility. Placebo-controlled studies of prucalopride in adults have shown that the medication not only increased the peristaltic amplitude but it also increases the number of swallows with complete peristalsis (44, 45). Paralleling the esophageal motility benefits, there are also significant improvements in gastric emptying in healthy controls and in patients with delayed gastric emptying (46, 47). While these physiology studies have been conducted in adults, there is evidence that prucalopride does improve upper tract symptoms in children; in a study of 71 children, 65% of patients had symptomatic improvement during the follow up period with the greatest improvements in patients presenting with typical reflux symptoms, vomiting and feeding difficulties (48). This combination of improved esophageal and gastric motility results in reduced acid exposure time in the esophagus, making prucalopride a promising novel therapy for pediatric reflux disease (47).

Another promising reflux therapy for pediatrics is intrapyloric Botulinum toxin (BoTox) injections. While studies have shown that BoTox injections may be beneficial in older children with nausea and vomiting, a study of 112 younger children (mean age: 2.9 ± 1.6 year) with symptoms of reflux, vomiting and feeding difficulties showed significant symptom improvement with pyloric injections of 6 units/kg (49, 50). Response was particularly good in patients with gastroesophageal reflux where 80% of these patients experienced some degree of symptomatic improvement (49). While these results are promising, additional studies are needed to determine the efficacy in patients with isolated GERD and where in the therapeutic algorithm BoTox may fall should larger studies determine if this experimental therapy is efficacious (49).

Another novel medication approach is the use of bile acid sequestrants to reduce the amount of bile reaching the esophagus as bile has been implicated as a cause for esophageal inflammation and, most recently, for more severe extraesophageal symptoms (51, 52). In a single adult study of 280 patients with refractory symptoms (defined as persistent symptoms 4 or more times a week despite once daily PPIs), patients were randomized to placebo or a twice-daily bile acid sequestrant. At high doses of sequestrant, there was significant improvement in heartburn symptoms over the 8-week trial (53). There are no studies of sequestrants in pediatrics but high concentrations of lung bile portend a worse clinical prognosis suggesting that there may be role for this class of medications to treat extraesophageal symptoms (51).

Finally, while not novel, neuromodulators have become part of the mainstay of reflux therapy to treat the pain symptoms not responsive to acid suppression (in the case of reflux hypersensitivity) or not associated with reflux events (in the case of functional heartburn). Selective serotonin reuptake inhibitors (e.g., citalopram, fluoxetine), tricyclic antidepressants (e.g., desipramine, imipramine), and GABA analogs (e.g., gabapentin) have been used to modulate both typical and even extraesophageal symptoms and are now part of the algorithms to treat persistent or severe symptoms (54–59). Their role in pediatric reflux is not known.

## Lifestyle and Trigger Modification

Dietary modification has long been a part of adult reflux guidelines but there is very little data of the impact of diet for symptomatic control. Mediterranean diets, alkaline water diets, high fiber diets, low fat, low calorie, vegetarian, gluten free, low-FODMAP diets have all been studied or proposed in very small trials or in retrospective reviews with varying success in reducing symptoms (60–64). Recent data suggests, however, that clinician-prescribed or self-prescribed diets can result in avoidant-restrictive food intake disorders (ARFID), particularly in adults and children with functional gastrointestinal disorders (65, 66). Therefore, with a lack of clear evidence for benefit, dietary restriction should be recommended very cautiously, particularly in teenagers. In pediatrics, studies of dietary modification has been focused on (1) thickened feeds for infants and children who are enterally fed with either gastrostomy or gastrojejeunal feeds; and (2) hypoallergenic formulas for infants. Both thickened feeds and hypoallergenic diets have shown benefit in reducing regurgitation and symptoms attributed to gastroesophageal reflux (67–69).

Another potential modifier for pediatric gastroesophageal reflux is sleep. Studies in adults have suggested that while acid suppression improved nocturnal sleep quality and reduces symptoms of gastroesophageal reflux disease, poor sleep quality can result in pain amplification making reflux symptoms worse (70, 71). While there are no comparable pediatric trials, sleep practices have been an integral part of pediatric well child assessments so represent an easily integrated area of intervention. Complementing regular sleep schedules, safe sleep positioning has been integral to pediatric well child care, particularly in infants. While supine sleeping is critical for sudden infant death prevention, pediatricians have long known, based on physiology studies, that left lateral positioning reduced gastroesophageal reflux events greater than right lateral positioning so positioning in the awake infant may offer symptom improvement (72, 73). Adult studies are just being published confirming the pediatric studies (74).

## Conclusion

The field of gastroesophageal reflux in children has changed dramatically with improved testing which has broadened the differential diagnosis to include motility disorders and functional disorders. With these new technologies, more critical information can be obtained at the index endoscopy. This earlier testing results in a faster time-to-definitive-diagnosis and allows for more precise tailoring of therapies beyond acid suppression. These diagnostic and therapeutic advances have resulted in a reimaging of existing algorithms including a new proposed pediatric algorithm (Figure 2). However, while the science has advanced significantly for pediatric patients, approval for pediatric medications beyond acid suppression are critically needed in order to get efficacious medications from the adult realm to the most vulnerable pediatric patients.

## Author Contributions

RR conceived of the ideas in this manuscript and wrote the draft in its entirety.

## Conflict of Interest

The author declares that the research was conducted in the absence of any commercial or financial relationships that could be construed as a potential conflict of interest.

## Publisher’s Note

All claims expressed in this article are solely those of the authors and do not necessarily represent those of their affiliated organizations, or those of the publisher, the editors and the reviewers. Any product that may be evaluated in this article, or claim that may be made by its manufacturer, is not guaranteed or endorsed by the publisher.
